# Critical factors driving spatiotemporal variability in the phytoplankton community structure of the coral habitat in Dongshan Bay, China

**DOI:** 10.3389/fmicb.2024.1355028

**Published:** 2024-02-16

**Authors:** Qianqian Zhou, Xu Dong, Jianjia Wang, Youyin Ye, Yanyan Yang, Peng Xiang, Yanghang Chen, Xinqing Zheng

**Affiliations:** ^1^Third Institute of Oceanography, Ministry of Natural Resources, Xiamen, China; ^2^Fujian Provincial Station for Field Observation and Research of Island and Coastal Zone in Zhangzhou, Zhangzhou, China; ^3^Observation and Research Station of Island and Coastal Ecosystem in the Western Taiwan Strait, MNR, Xiamen, China

**Keywords:** phytoplankton, community structure, spatiotemporal variability, coral habitat, current, nutrient

## Abstract

This study investigated the spatiotemporal distribution of the phytoplankton in the coral habitat of Dongshan Bay (China), along with critical factors affecting the distribution, during June, August, and December 2022. Phytoplankton abundance in Dongshan Bay exhibited considerably temporal variation, peaking in June 2022, gradually decreasing thereafter, and reaching its lowest point in December 2022. The abundance of bottom-layer phytoplankton consistently exceeded that of the surface layer throughout all seasons. The average phytoplankton abundance in the coral habitat of Dongshan Bay was lower than that in non-coral habitat areas. Fluctuations in the Zhangjiang River and coastal upwelling influenced the diversity and community structure of the phytoplankton. Critical factors causing spatiotemporal variability in phytoplankton community structure included nutrient concentrations and seawater temperature. Nutrients played key roles in influencing various phytoplankton groups. Dominant diatom species, such as *Thalassionema nitzschioides* and *Thalassiosira diporocyclus*, were positively correlated with ammonia nitrogen, seawater salinity, coral cover, and the number of coral species present. In winter, *Calanus sinicus* exhibited a negative correlation with harmful algal bloom species. Additionally, it was found that both in the coral habitat and surrounding open sea, currents, nutrients, and zooplankton may play crucial roles in determining the spatiotemporal variability in the phytoplankton community structure.

## 1 Introduction

Phytoplankton, as a main food source for marine zooplankton, plays an important role in the energy flow and nutrient cycling of marine ecosystem. Variability in phytoplankton community structure can have negative ecological impacts on marine ecosystems (See et al., 2005), such as leading to harmful algal blooms (Paerl, 1988), which provide a sensitive early warning for climate-driven perturbations in marine ecosystems (Hallegraeff, 2010), and affecting the quality of food sources for outbreaking marine larval organisms such as the Crown-of-Thorns Starfish (Peng C. et al., 2023).

Coral reefs, among the most diverse ecosystems on Earth (Zhao et al., 2006; Galand et al., 2023), can utilize heterotrophic food sources and photosynthate transferred from their symbiotic dinoflagellate (Zheng et al., 2021). On crossing the coral reefs, oceanic water becomes enriched with various detritus, including organic particles, aggregates, coral mucus and more, which in turn provides a substantial food source for other organisms (Sournia and Ricard, 1976). The sensitivity of phytoplankton to variations in water quality parameters enables them to indirectly serve as indicators of the health status of coral reefs (Limbu and Kyewalyanga, 2015).

Recently, there have been multiple studies on the phytoplankton community structure of coral reefs in various bays and oceans worldwide (Sournia and Ricard, 1976; Jales et al., 2022; Şahin et al., 2022; Zhang J. et al., 2022; Soondur et al., 2023). Some research findings (Soondur et al., 2023) indicate that diatoms and dinoflagellates are more abundant in non-degraded coral reefs, whereas cyanobacteria exhibit higher densities in degraded coral reefs. Non-degraded coral reefs are more suitable for the growth and reproduction of phytoplankton compared to degraded coral reefs. The phytoplankton population in coral reef areas is rich in species, with diatoms constituting the main group, followed by highly diversified dinoflagellates. The abundance of phytoplankton tends to be the highest nearshore (Sournia and Ricard, 1976; Şahin et al., 2022). Phytoplankton composition in an atoll environment is primarily associated with nutrient salts (Jales et al., 2022). Shallow-water coral reefs will be affected more often by episodic smothering conditions caused by harmful algal blooms. For example, the green *Noctiluca* results in a reduction in the dissolved oxygen content in seawater, leading to coral mortality (Samiyappan et al., 2023).

Biogeochemistry plays a critical role in the evolution of coral reefs with notably impacts on the relationship between corals and phytoplankton (Zhang J. et al., 2022). For instance, regional variability of seawater *p*CO_2_ in Dongshan’s coral habitats depends on the organic carbon metabolism of marine phytoplankton, especially diatoms (Dong et al., 2023). The spatial variation in phytoplankton abundance among six coral atolls in the central South China Sea is significant across different regions of the atolls. The highest phytoplankton abundance observed in Huangyan Atoll indicates potential anthropogenic eutrophication (Ke et al., 2018). The micro-phytoplankton will serve as an indicator for the health status of the coral ecosystem from a biological perspective (Soondur et al., 2023). A ubiquitous high phytoplankton biomass in the waters near coral reefs is a steady source of organic nutrients to the coral reef ecosystems (Zhou et al., 2023). The variability in structure and function of bacterioplankton communities between areas of oyster culture and coral reefs is mainly driven by salinity and ammonium (Tong et al., 2021). The environmental parameters of Dongshan Bay vary in different seasons (Chen et al., 2023), but there is no available data to demonstrate how phytoplankton communities contribute to these varieties.

Current plays a significant role in the sea by transporting nutrient-rich water toward the bay, leading to high phytoplankton abundance and primary productivity (Ma et al., 2021). Influenced by the current, environmental changes such as temperature variations, nutrient availability, and light often result in significant fluctuations in phytoplankton species, which in turn affect the community structure of phytoplankton. Several studies (Silva et al., 2009; Du and Peterson, 2014) have shown that phytoplankton in coastal ecosystems undergoes seasonal or interannual changes in community structure influenced by current. Diatoms dominate during the upwelling season, and as the upwelling weakened, there was a transition from diatoms to dinoflagellates.

In this study, we investigated the spatiotemporal distribution of the phytoplankton community structure in the coral habitat of Dongshan Bay, China, in June, August, and December 2022. The aims of this study include, (1) to investigate the characteristics of phytoplankton community structure in coral habitat; (2) to better understand the relationship between the phytoplankton community structure in the coral habitat and abiotic and biological factors; (3) to explore the critical factors driving variability in the phytoplankton community structure in Dongshan Bay; (4) to determine whether the distribution of corals influences the community structure of phytoplankton. The results of the study provide essential information about the phytoplankton community structure in Dongshan Bay’s coral habitat, contributing to its conservation and management.

## 2 Materials and methods

### 2.1 Study areas

**Figure 1:**
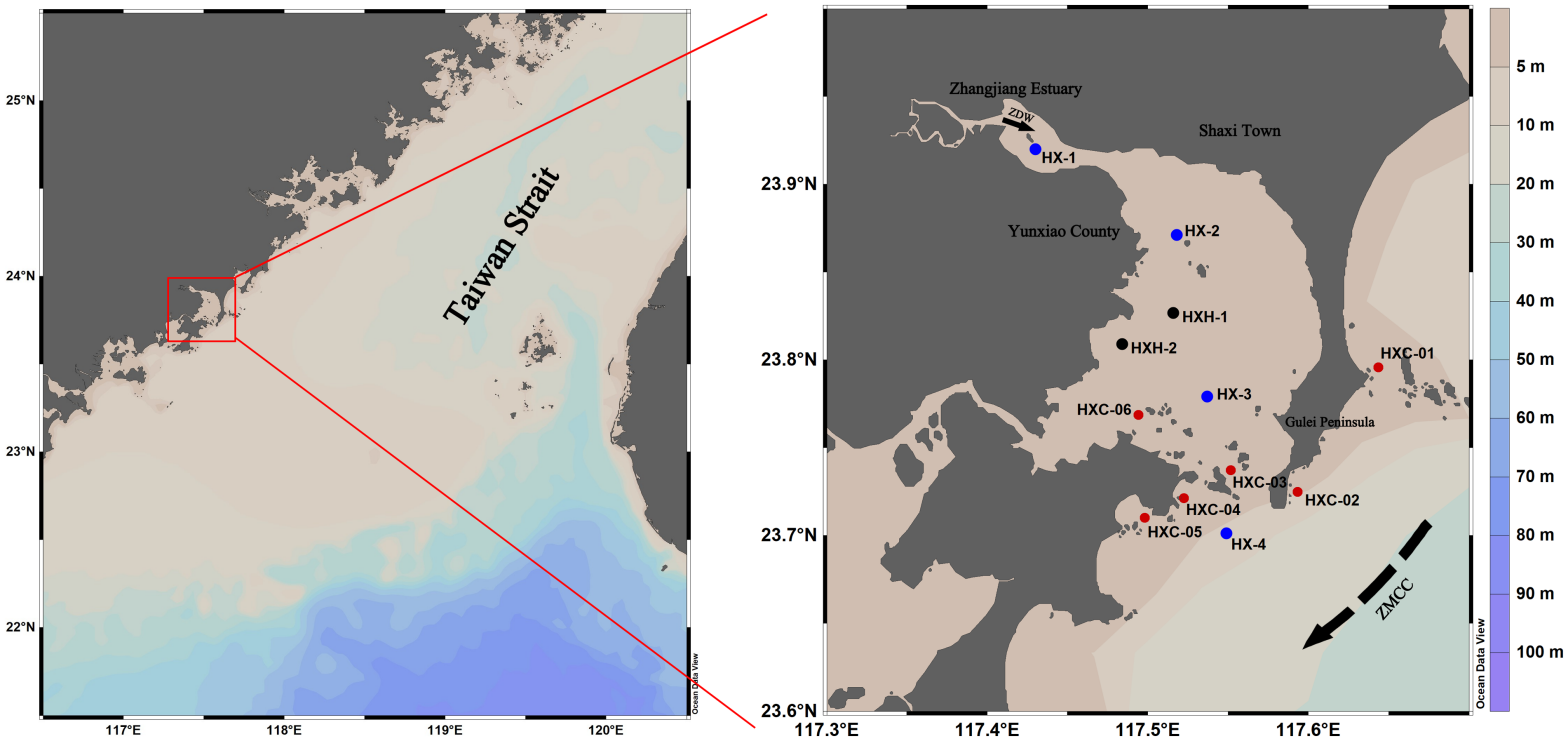
Geographic location of the investigated stations in Dongshan Bay, China (black sampling sites are not included in August; blue sampling sites are non-coral habitat stations; red sampling sites are coral habitat stations). In the right figure, the solid black arrow indicates the Zhangjiang River Diluted Water (ZDW); the dotted black arrow indicates Zhe-Min Coastal Current (ZMCC).

Dongshan Bay, is located on the western coast of the southern entrance to the Taiwan Strait. It extends irregularly into the mainland and has a plowshare-like shape, with the estuary of the Zhangjiang River at its bayhead (Figure 1). This narrow inlet bay has strong isolative characteristics. It is influenced by solar radiation, the Taiwan Strait, and a monsoonal circulation due to its location within a low-latitude belt (Wen, 2017). Within Dongshan Bay is the Dongshan Coral Provincial Nature Reserve, which is home to coral communities that represent the northernmost distribution of reef-building corals along the mainland coast of China. The reserve boasts a rich diversity of species. 12 stations in Dongshan Bay were investigated in June, August, and December 2022 (Figure 1).

### 2.2 Sampling and environmental factor analysis

Temperature (T), salinity (S), dissolved oxygen (DO) and pH of seawater were measured *in situ* using a YSI EXO2 multiparameter water quality sonde (YSI Inc., Ohio, United States). For nutrient analysis, 0.5 L of seawater samples were filtered through pre-cleaned 0.45 μm cellulose acetate membranes, which were then preserved at 4°Cand immediately analyzed upon return to the laboratory. Concentrations of ammonium (NH_4_^+^), nitrate (NO_3_^−^), nitrite (NO_2_^−^), phosphate (PO_4_^3−^), and silicate (SiO_3_^2−^) were determined using colorimetric methods as specified in the Oceanographic Survey-Part 4: Survey of Chemical Parameters in Sea Water (General Administration of Quality Supervision, 2008).

To examine the phytoplankton community structure of the coral habitat in Dongshan Bay, we collected 500 mL water samples using Niskin bottles (0.50 L) from both the surface and bottom layers. As per the Specifications for Oceanographic Survey-Part 6: Marine Biological Survey, it is recommended to collect surface and bottom water samples when the water depth exceeds 5 meters. However, when the water depth is less than 5 meters, collecting only surface water samples is sufficient (General Administration of Quality Supervision, 2008). Samples were preserved with 1% acetic Lugol’s iodine solution, concentrated to a final volume of 10 mL after sedimentation, and stored in the dark until they were counted. For permanent storage, 1–2 drops of formaldehyde were added to each sample.

Zooplankton samples were collected by vertically towing a net with dimensions of 145 cm in length, 50 cm in dimeter, and a mesh size of 505 μm from near the bottom of the sea surface. After collection, all samples were preserved in a 5% formalin-seawater solution and subsequently analyzed in the laboratory.

The status of the coral community in 2022 was surveyed using the line intercept transect method (Zheng et al., 2021). Videos were recorded slowly above a 50 m transect tape. Only live scleractinian corals within the belt transect and directly under the tape at 10 cm intervals were identified to the species level. The data and video images were used to determine the percent cover of different substrate types and various benthic communities, including scleractinian corals, soft corals, and macroalgae. Coral community surveys were conducted only in June and August, except for station HXC-06 which did not conduct a coral community survey in June.

### 2.3 Phytoplankton and zooplankton identification and counting

Every phytoplankton species was identified and counted with a phytoplankton enumeration chamber (0.1 mL, 20 × 20 mm) using an inverted microscope (Leica DMi8A, Germany) at 100–400× magnification. Initially unknown species were later identified using a FEI Quanta450 (United States) transmission electron microscope. To prepare for identification, diatom cells were cleaned using H_2_SO_4_ and rinsed with distilled water until neutral. The dinoflagellate cells were dehydrated using an ethanol series (10, 30, 50, 70, 90%), followed by three times in 100% ethanol, with each step lasting 10 min. Afterward, the cells were critically dried, coated with gold using a sputter-coater, and examined using a scanning electron microscope.

Zooplankton were identified and enumerated using a stereoscopic microscope (Leica M165 FC), with smaller diagnostic features being investigated using a Leica Inverted Microscope (Model DMi8A).

### 2.4 Statistical analysis

In this study, dominant species in phytoplankton community were defined as those with a species dominance greater than 0.02 (Xu and Chen, 1989). The Shannon-Wiener Index (*H′*), Pielou Evenness Index (*J’*), and Margalef Richness Index (*d*) were used the characterize the phytoplankton community structure; their calculation Eqs. 1–4 are as follows:

Shannon-Wiener Index (Shannon and Wiener, 1949)


(1) $$H^{\prime }=\overset{s}{\underset{i=1}{\sum }}\mathrm{Pi}\log_{2}\mathrm{Pi}$$

Pielou Evenness Index (Pielou, 1966)


(2) $$J^{\prime }=H^{\prime }/\log_{2}S$$

Margalef Richness Index (Margalef, 1958)


(3) $$d=\left(S-1\right)/\ln N$$

Species Dominance (Xu and Chen, 1989)


(4) $$Y=n_{i}/N\times f_{i}$$

S represents the sum of phytoplankton species observed in the community, N represents the total phytoplankton abundance of the community, n_i_ represents the cell abundance of No. i species in the community, and fi represents the frequency of the No. i species occurring at each station in the community.

The survey data were visualized using Ocean Data View (5.3.0) software to create a distribution map of phytoplankton abundance. Due to the limited number of stations with a water depth greater than 5 meters, bottom water samples were collected. Consequently, the spatiotemporal distribution of phytoplankton abundance considers the average abundance of both the surface and bottom layers. To analyze the phytoplankton community structure, cluster analysis was performed using Primer 5.29 software. Additionally, the ‘circlize’ package in R (v4.3.1) was used to visualize the circos plot of spatiotemporal variability in the average abundance and species numbers of phytoplankton. The relationship between phytoplankton community structure and the major environmental factors (nutrients, corals, zooplankton) was analyzed using CANOCO software. Environmental factors were used as explanatory variables, and dominant species with a dominance level exceeding 0.02 in at least one sample were included in the analysis. Species data and environmental parameters were log_10_ (x + 1) transformed for subsequent analysis to achieve a normal distribution. Detrended correspondence analysis was used to determine the appropriate methods for the species data. Subsequently, ordination analysis was conducted, and ordination diagrams were generated to analyze the correlation between various species and environmental factors.

## 3 Results

### 3.1 Community composition and structure of phytoplankton

**Figure 2:**
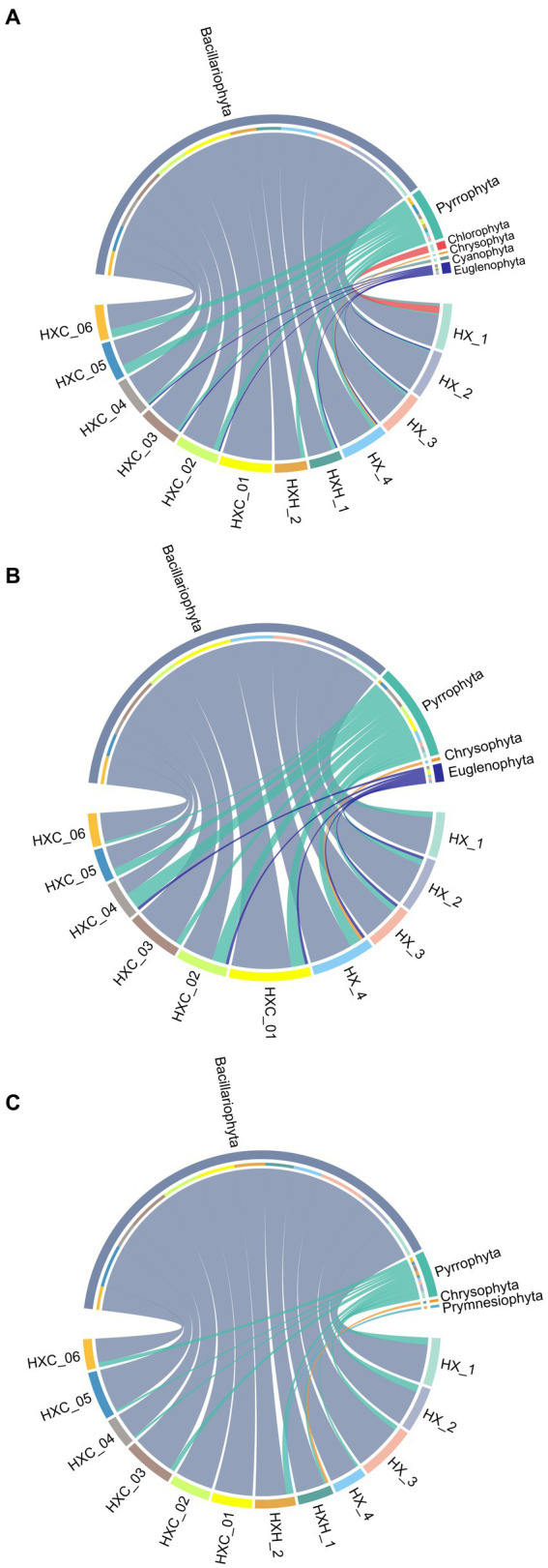
Spatial variations of phytoplankton species number **(A)** June; **(B)** August; **(C)** December.

**Figure 3:**
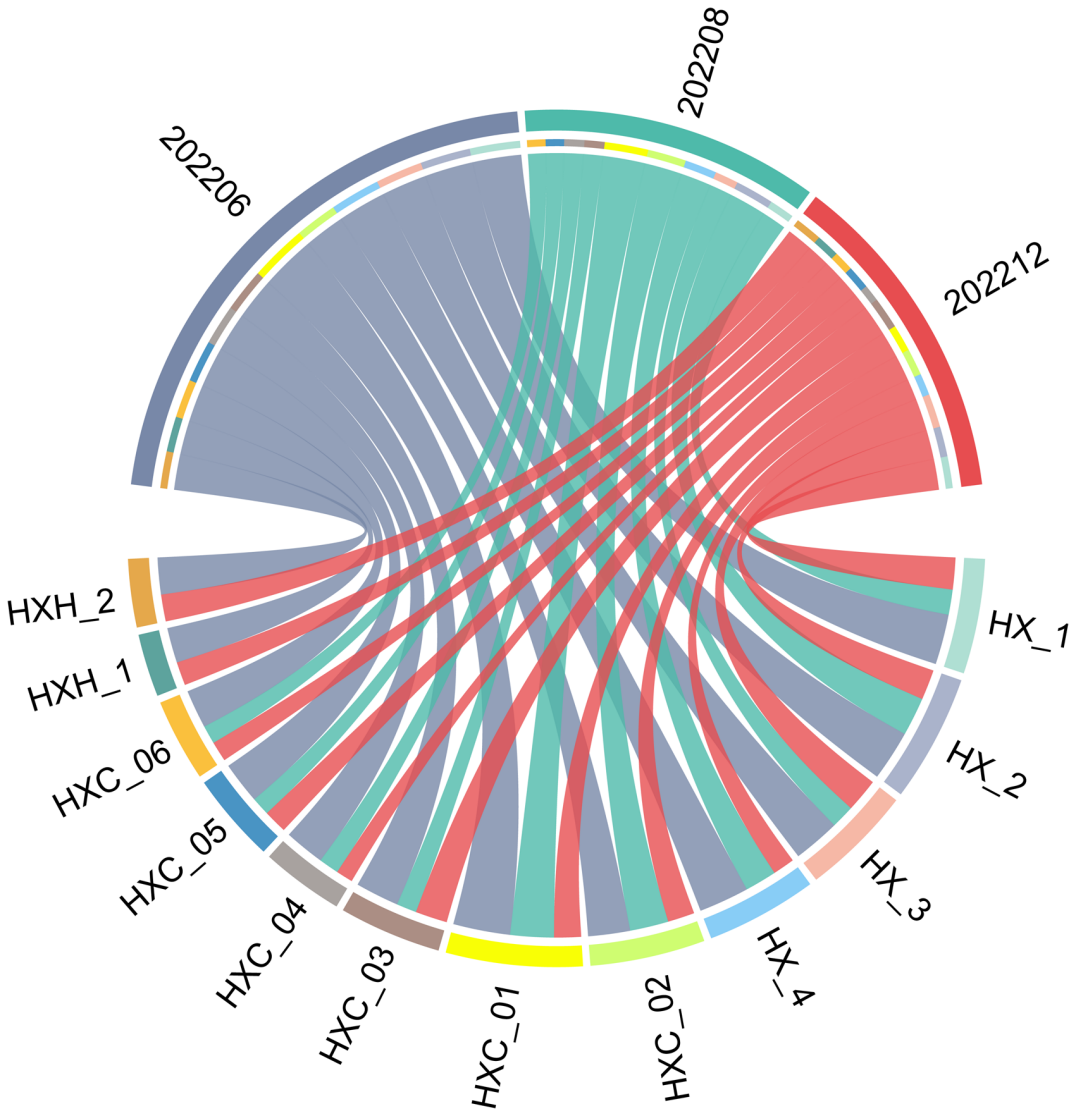
Temporal variation of phytoplankton species number in Dongshan Bay.

During the study period, a total of 135 phytoplankton species were identified in Dongshan Bay, belonging to 7 phyla and 63 genera. The phytoplankton community was composed of 101 species of Bacillariophyta (74.81%), 22 species of Pyrrophyta (16.30%), five species of Chlorophyta (3.70%), three species of Chrysophyta (2.22%), two species of Cyanophyta (1.48%), one species of Prymnesiophyta (0.74%), and one species of Euglenophyta (0.74%). In June (Figure 2A), the dominant phylum was Bacillariophyta with 78 species (76.47%), followed by Pyrrophyta with 15 species (14.71%), Chlorophyta with five species (4.90%), Cyanophyta with two species (1.96%), Chrysophyta with one species (0.98%), and Euglenophyta with one species (0.98%). In August (Figure 2B), Bacillariophyta remained the dominant phylum with 55 species (79.71%), followed by Pyrrophyta with 12 species (17.39%), Chrysophyta with one species (1.45%), and Euglenophyta with one species (1.45%). In December (Figure 2C), Bacillariophyta was still the dominant phylum with 66 species (89.19%), followed by Pyrrophyta with six species (8.11%), Chrysophyta with one species (1.35%), and Prymnesiophyta with one species (1.35%). The highest species number was observed in June 2022 with 102 species, while the lowest species number was recorded in August 2022 with 69 species (Figure 3). The temporal variation of diatom (Bacillariophyta) species number mirrored that of the total phytoplankton. Dinoflagellates (Pyrrophyta) exhibited the lowest diversity in December 2022, but diatoms consistently dominated as the primary phylum throughout the study period. The complete list of phytoplankton species can be found in the Supplementary Table S1.

The dominant species of phytoplankton in Dongshan Bay were primarily diatoms, with notable seasonal variations. These diatoms, which formed chain-like structures, included red tide causing species such as *Chaetoceros curvisetus*, *Skeletonema costatum*, *Thalassionema nitzschioides*, *Asterionella japonica*, and others. In August, the dinoflagellate *Scrippsiella trochoidea* also emerged as one of the dominant species. The abundance and species dominance of phytoplankton in Dongshan Bay are presented in Table 1. In June, *C. curvisetus* had the highest cell abundance, while *T. nitzschioides* had the lowest abundance in August. In terms of species dominance, *Pseudo-nitzschia delicatissima* had the highest dominance in August 2022.

**Table 1:** Temporal variation of phytoplankton dominant species in Dongshan Bay.

Season	Dominant species	Abundance (10^4^cells/L)	Species dominance
Jun. 2022	*Chaetoceros curvisetus*	12.91	0.32
	*Skeletonema costatum*	8.95	0.22
	*Asterionella japonica*	1.75	0.04
	*Chaetoceros affinis*	1.5	0.04
	*Pseudo-nitzschia pungens*	1.21	0.03
Aug. 2022	*Pseudo-nitzschia delicatissima*	5.19	0.39
	*Thalassionema nitzschioides*	4.35	0.33
	*Scrippsiella trochoidea*	0.63	0.05
	*Rhizosolenia sinensis*	0.67	0.02
Dec. 2022	*Thalassionema nitzschioides*	0.51	0.18
	*Melosira sulcata*	0.43	0.12
	*Thalassiosira rotula*	1.64	0.06
	*Skeletonema costatum*	0.56	0.05

### 3.2 Cluster analysis and ecological indices of phytoplankton

**Figure 4:**
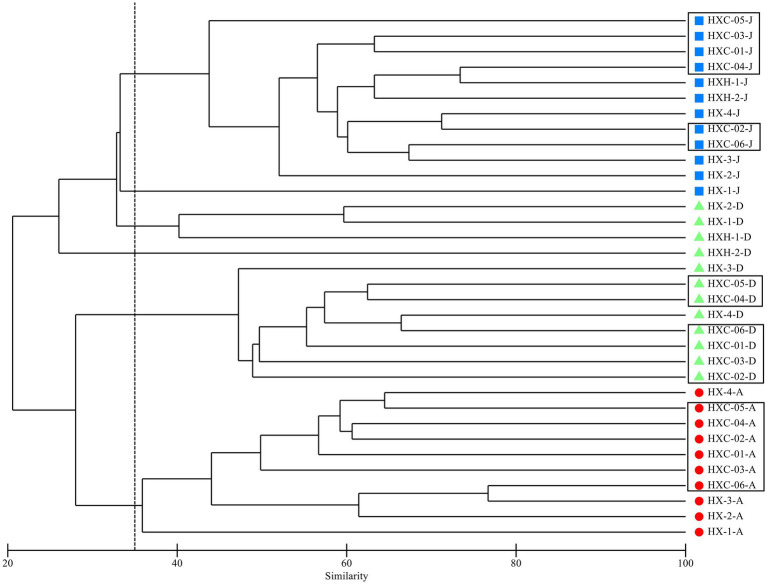
Dendrogram of cluster analysis of phytoplankton assemblage collected in Dongshan Bay.

The cluster analysis of the phytoplankton community structure in Dongshan Bay (Figure 4) revealed significant differences in spatiotemporal distribution. The community can be clearly divided into four groups at 30% similarity level, with noticeable clustering observed in June and August, indicating obvious seasonal aggregation. In December, two groups emerge: one in the near estuarine and nearshore non-coral habitat areas, and the other in the outer part of Dongshan Bay, within the coral habitat area. In both June and August, at the 40% similarity level, there was a significant difference between station HX-1 and other sampling stations. However, in December station HX-3 and HX-4 distinctly cluster with the coral habitat area.

**Figure 5:**
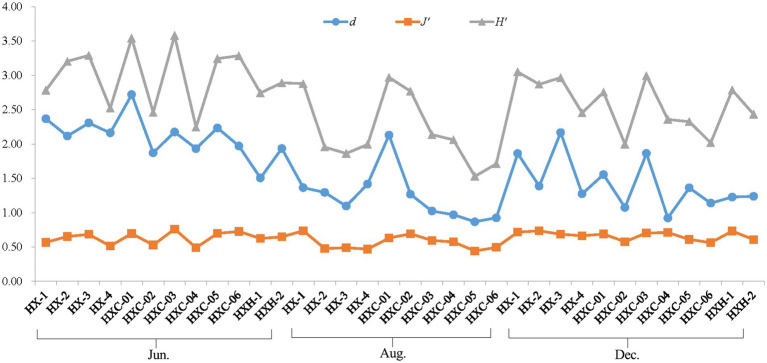
Ecological indices of phytoplankton in Dongshan Bay.

The phytoplankton Shannon-Wiener Index (*H′*) and Pielou Evenness Index (*J’*) were highest in June at station HXC-03, which is located in the coral habitat area. Similarly, station HXC-01 had the highest Margalef Richness Index (*d*) in June. Conversely, the lowest values for phytoplankton *H′*, *J’*, and d throughout the year were recorded at station HXC-05 in August (Figure 5). The average values of *H′* and *d* were highest in June, followed by December, and lowest in August for the entire year of 2022. On the other hand, *J’* was highest in December, followed by June, and lowest in August. In June and August, *H′*, *J’*, and *d* were higher in coral habitats compared to non-coral habitats (Table 2). However, in December, the trend was reversed, with non-coral habitats showing higher values for *H′*, *J’*, and *d* compared to coral habitats. In both August and December, the total number of phytoplankton species in coral habitat areas was higher than in non-coral habitat areas. Conversely, in June, the total number of phytoplankton species in non-coral habitat areas was higher than in coral habitat areas.

**Table 2:** Ecological indices of phytoplankton in different areas (mean ± S.D.).

	Coral Habitat Area			Non-Coral Habitat Area		
	Jun. 2022	Aug. 2022	Dec. 2022	Jun. 2022	Aug. 2022	Dec. 2022
S	68	54	74	84	50	60
*d*	2.15 ± 0.31	1.20 ± 0.48	1.32 ± 0.35	2.07 ± 0.31	1.29 ± 0.14	1.53 ± 0.39
*J’*	0.65 ± 0.11	0.57 ± 0.09	0.64 ± 0.07	0.62 ± 0.06	0.54 ± 0.13	0.69 ± 0.05
*H′*	3.06 ± 0.57	2.20 ± 0.57	2.41 ± 0.40	2.91 ± 0.29	2.17 ± 0.47	2.76 ± 0.26

S stands for total species; d stands for Margalef Richness Index; J′ stands for Pielou Evenness Index; H′ stands for Shannon-Wiener Index.

### 3.3 Abundance of phytoplankton

**Figure 6:**
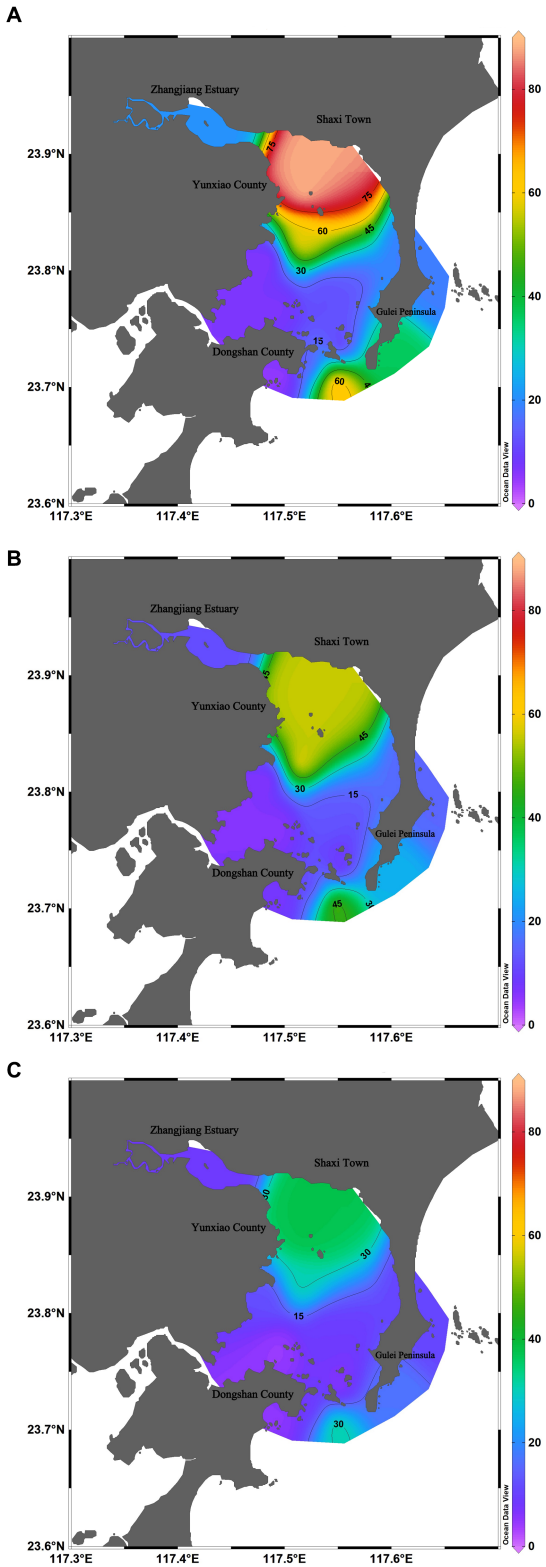
Spatiotemporal distribution of phytoplankton abundance in Dongshan Bay **(A)** June; **(B)** August; **(C)** Decmber.

Among three surveys, in June 2022, Dongshan Bay exhibited the highest average abundance of phytoplankton, with an average abundance of 28.64 × 10^4^cells/L. The phytoplankton abundance at station HX-2 recorded the highest value, reaching 88.80 × 10^4^cells/L, primarily due to the presence of *C. curvisetus* and *S. costatum*. The zone with high phytoplankton abundance was mainly concentrated in the Zhangjiang estuary and the open waters of Dongshan Bay, while the adjacent coastal stations showed lower phytoplankton abundance (Figure 6A).

In August 2022, the average abundance of phytoplankton reached 12.66 × 10^4^cells/L. The highest value was recorded at station HX-4, reaching 32.45 × 10^4^cells/L, with the primary contributing species being *P. delicatissima* and *T. nitzschioides*. The zone with high phytoplankton abundance was only concentrated in the open waters of Dongshan Bay, while the adjacent coastal stations showed lower phytoplankton abundance (Figure 6B).

In December 2022, the average abundance of phytoplankton was the lowest among the three surveys, with an average abundance of 1.47 × 10^4^cells/L. The highest value was recorded at station HX-2, reaching 2.42 × 10^4^cells/L, primarily represented by *S. costatum*, *Thalassiosira rotula*, and *C. curvisetus*. The zone with high phytoplankton abundance was only concentrated on the nearshore of the inner bay (Figure 6C).

**Figure 7:**
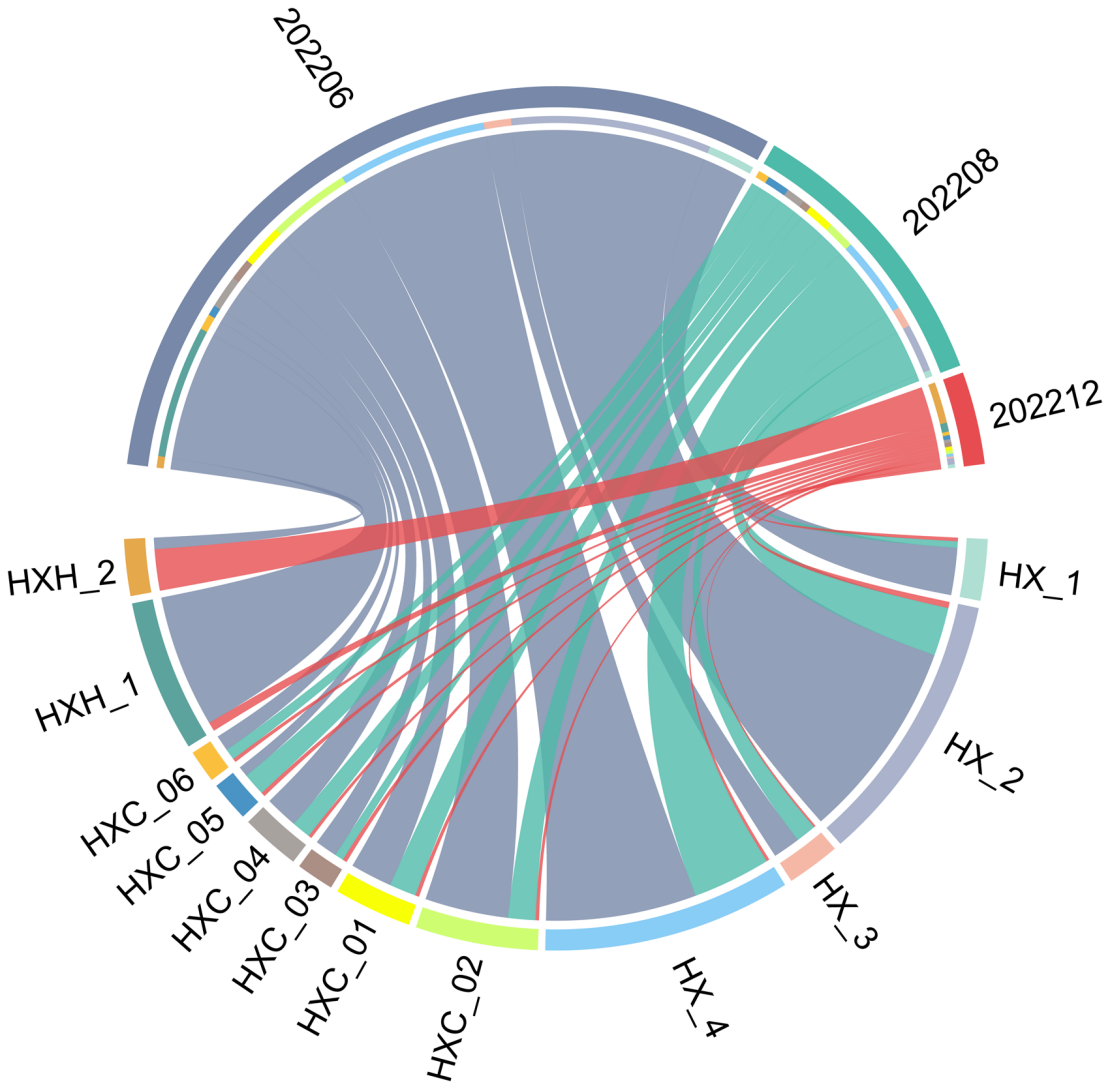
Circos plot of temporal variation of phytoplankton abundance in Dongshan Bay.

Temporal variation in phytoplankton abundance in Dongshan Bay was observed, as shown in Figure 7. The abundance reached its peak in June 2022 and gradually decreased thereafter, reaching its lowest point in December 2022. Furthermore, the average abundance of phytoplankton in the coral habitat area of Dongshan Bay was found to be lower compared to non-coral habitat areas.

### 3.4 Environmental factors

**Figure 8:**
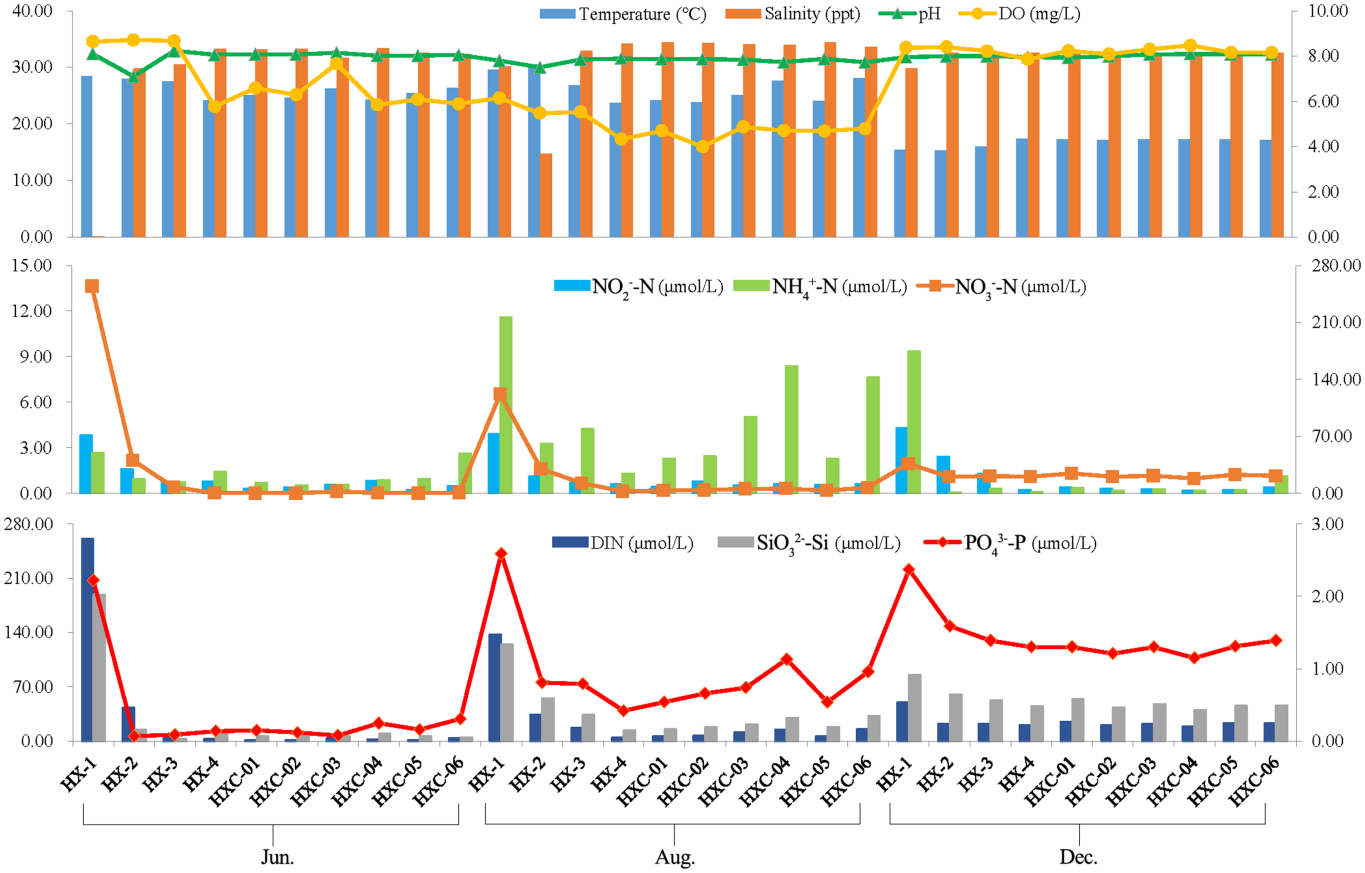
Spatiotemporal variability of chemical factors in Dongshan Bay (The left vertical axis represents the values of the bar chart, while the right vertical axis represents the values of the line chart.).

The spatiotemporal variation of chemical factors in Dongshan Bay was presented, as shown in Figure 8. The water temperature ranged from 15.28 to 30.55, salinity ranged from 0.10 to 34.43, and pH ranged from 7.50 to 8.15. The salinity levels in the coral habitat area were higher compared to the non-coral habitat, with station HX-1 recording a salinity of only 0.1 in June. Temporal variability was observed in the water temperature of Dongshan Bay, with the lowest temperature occurring in December. On the other hand, salinity showed notable spatial variability in the bay, with the lowest salinity observed in nearshore stations like HX-1 and HX-2. The range of dissolved oxygen (DO) in Dongshan Bay was 4.00 to 8.64 mg/L, with the lowest value recorded in August, higher values in June and highest in December. However, the concentration of ammonia nitrogen (NH_4_^+^-N) ranged from 0.07 to 11.58 μmol/L, which the highest value occurring at station HX-1 in August. Nitrate nitrogen (NO_3_^−^-N) ranged from 0.15to 254.62 μmol/L, with the highest value occurring at station HX-1 in June. Nitrite nitrogen (NO_2_^−^-N) exhibited notable spatial variability in Dongshan Bay, with higher values in the nearshore area compared to the offshore. Dissolved inorganic nitrogen (DIN) ranged from 1.10 to 261.11 μmol/L, SiO_3_^2−^-Si ranged from 2.79 to 188.30 μmol/L and PO_4_^3−^-P ranged from 0.07 to 2.37 μmol/L. DIN, SiO_3_^2−^-Si, and PO_4_^3−^-P in the estuary area were higher than those in other stations.

**Figure 9:**
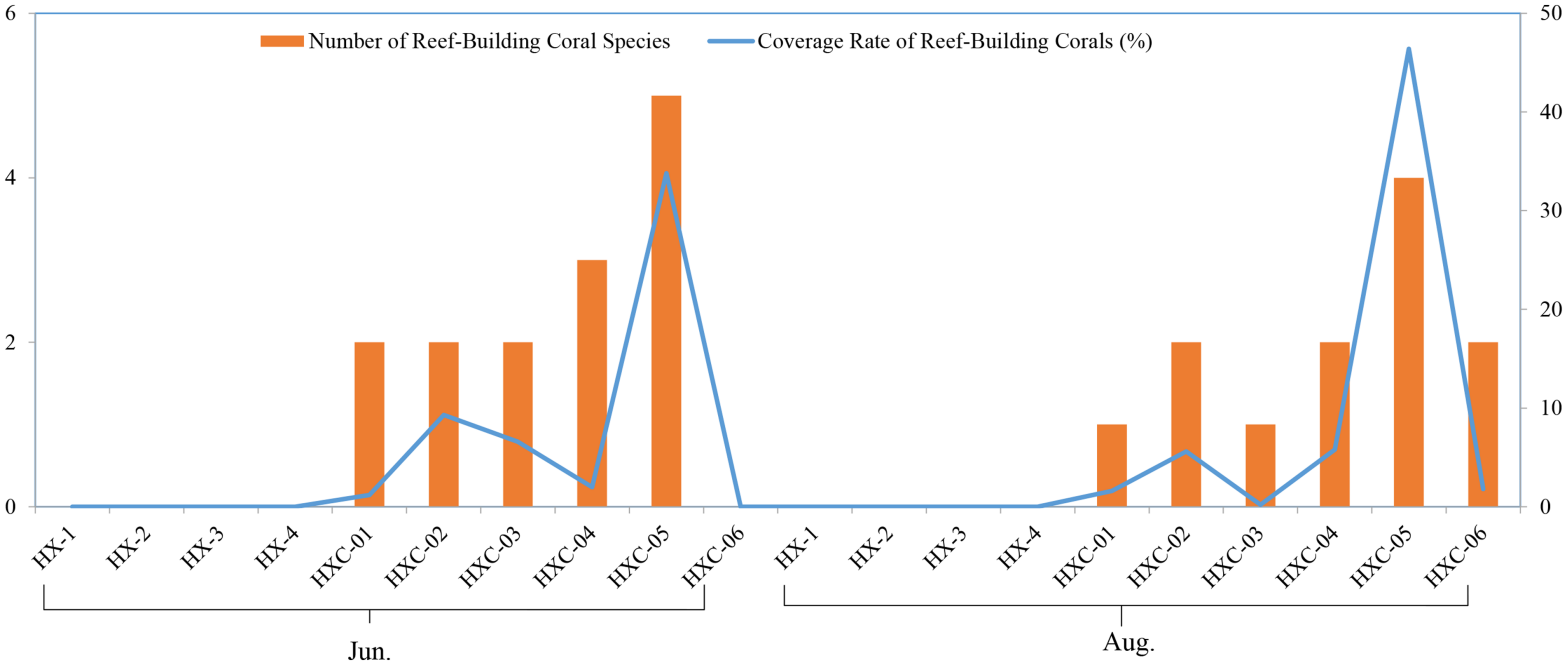
Spatiotemporal variability of coral community in Dongshan Bay (The left vertical axis represents the values of the bar chart, while the right vertical axis represents the values of the line chart.).

The number of reef-building coral species was higher at the offshore station HXC-04 and HXC-05. Additionally, the coverage rate of reef-building coral was higher in August compared to June (Figure 9).

The ecological groups of zooplankton in Dongshan Bay mainly consisted of nearshore species. These nearshore species were predominantly represented by nearshore warm-water species and nearshore eurythermal species. The former group includes species such as *Lucifer hanseni*, *Oikopleura dioica*, *Diphyes chamissonis*, *Lensia subtiloides,* and others. The latter group comprises species like *Acartia pacifica* and *Pleurobrachia globosa*. Furthermore, some nearshore warm-temperate species like *Calanus sinicus* and *Pseudeuphausia sinica* are also present. In June and August, the dominant species include *Acartia pacifica*, *Sagitta enflata* and *Dolioletta gegenbauri*, while *Subeucalanus subcrassus* emerges as a dominant species in August and December.

## 4 Discussion

### 4.1 The response of phytoplankton community structures to current

The diversity and community structure of phytoplankton are influenced by fluctuations in water systems, and coastal upwelling (Du and Peterson, 2014; Ye et al., 2017; Zhong et al., 2019; Wilson et al., 2021; Peng P. et al., 2023). The effects of upwelling on phytoplankton abundance and community composition result from the combined influences of physical transport mechanisms and the impacts of physiological bottom-up control (Ma et al., 2021). Dongshan Bay, located on the western coast of the southern entrance to the Taiwan Strait, experiences the impact of a horizontal residual circulation system characterized by the dilution of freshwater from the Zhangjiang River estuary and counterclockwise circulation within the bay (Zheng et al., 2009). The water exchange capacity in Dongshan Bay is the strongest at the bay mouth, but weaker at the top of the bay (Qin et al., 2020).

Due to its location at the mouth of the Zhangjiang River near the downstream estuary area, HX-1 experiences a noticeable influx of fresh water from the river (Lang, 2021). Consequently, the cluster analysis indicates that station HX-1 belongs to a distinct phytoplankton group in comparison to the other stations in June (Figure 4). The investigation of phytoplankton community structure revealed the presence of numerous brackish and even freshwater species, such as *Melosira sulcata*, *Pediastrum duplex*, *Scenedesmus*, and *Merismopedia*, were found. The stations of the coral habitat area are located near the upwelling zone of Dongshan Bay, also known as the Minnan Upwelling Region (Xiao et al., 2002). The upwelling zone begins to form around June but initially with relatively weak intensity. By July, accompanied by an increase in intensity, the upwelling area expands southeastward (Li and Li, 1989; Xiao et al., 2002). Consequently, only HXC-01 and HXC-03 among the stations in the coral habitat share a common phytoplankton group, whereas the estuarine and other areas do not exhibit distinct phytoplankton groups; instead, a discernible blending of phytoplankton communities is observed.

In August, the upwelling region further enlarges, extending southeastward to potentially connect with the upwelling zone northwest of the shallow banks near Taiwan. Alternatively, it may merge southward with the upwelling zone off the eastern coast of Guangdong. During this period, both the distribution range and intensity reach their maximum. In September, the upwelling begins to decline (Chen et al., 1982; Hong et al., 1991). Station HX-1 continues to be considerably influenced by diluted water from Zhangjiang River, which is also associated with a distinct phytoplankton group. On the other hand, station HX-4, situated near the open sea and the coral habitat area (HXC01-HXC05), and it shares the same phytoplankton groups as other coral habitats (Figure 4).

In December, as the dilute water from the Zhangjiang River recedes, seawater flows into the downstream area of the river, resulting in increased water exchange within the bay (Lang, 2021). Consequently, there is a significant aggregation of phytoplankton groups at the estuarine stations HX-1, HX-2 and the inner bay stations HXH-1 and HXH-2. The Zhe-Min coastal current, as the primary current system on the western side of the Taiwan Strait, flows southward along the west coast of the strait during winter, driven by the strong northeast monsoon. Its influence extends as far south as the sea near Dongshan to Nan’ao Island (Wu, 1982, 1983; Wan et al., 2013; Wang et al., 2018), impacting the coral habitat area of Dongshan Bay. Consequently, the coral survey stations, along with HX-3 and HX-4, exhibit a shared phytoplankton group (Figure 4).

Previous studies have revealed differences in the dominant phytoplankton groups between river plume water and upwelled water, with diatoms identified as the dominant group in upwelled water (Tong et al., 2023). In the present study, station HX-1 was considerably influenced by river plume water, leading to distinct phytoplankton assemblages in comparison to the coral habitat area of Dongshan Bay. The phytoplankton community in the Bay is predominantly characterized by diatoms, serving as the primary functional group. Its higher diversity of phytoplankton assemblages is mainly due to the influence of the currents, which may carry species from different origins, such as brackish, tropical, warm-temperate, and cosmopolitan species.

### 4.2 The response of phytoplankton abundance variation to chemical factors

River plumes and coastal upwelling systems provide rich nutrient habitats for plankton (Tong et al., 2023). The nutrient enrichment facilitated by upwelling drives alterations in the community structure of phytoplankton, biomass, physiology, size fraction, and other relevant parameters (Wang et al., 2016; Du and Peterson, 2018; Yang et al., 2019; Liu et al., 2022; Mo et al., 2023).

**Figure 10:**
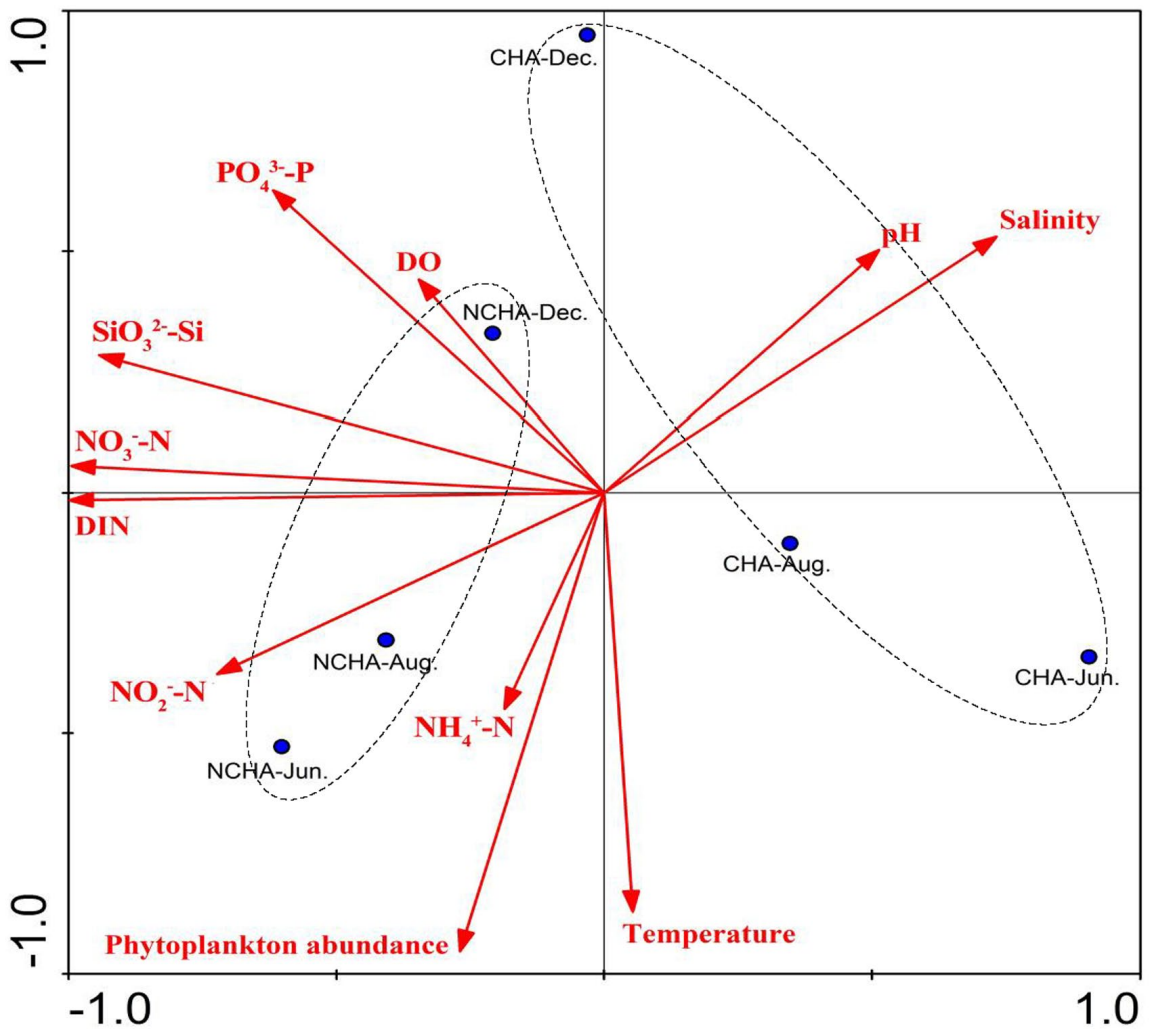
Principal Component Analysis (PCA) biplot analysis for phytoplankton abundance with physico-chemical parameters in Dongshan Bay. (NH_4_, ammonia nitrogen; DIN, dissolved inorganic nitrogen; PO_4_, phosphate; SiO_3_, silicate; NO_3_, nitrate; NO_2_, nitrite; DO, dissolved oxygen; CHN, Coral Habitat Area; NCHA, Non-Coral Habitat Area).

In the present study, the phytoplankton abundance in the non-coral habitat area was consistently higher compared to the coral habitat area. This finding was positively correlated with nutrients such as nitrogen and silicon (Figure 10), which supports previous research indicating that the waters of the coral atolls have low phytoplankton biomass and nutrient concentrations (Ke et al., 2018). However, Soondur et al. (2023) reported their findings, suggesting that the non-degraded reef area had higher phytoplankton abundance compared to the degraded area. We believe that this discrepancy may be due to the proximity of the non-coral habitat area to the Zhangjiang River estuary, which experiences severe eutrophication caused by frequent human activities in Dongshan Bay (Chen et al., 2014).

**Figure 11:**
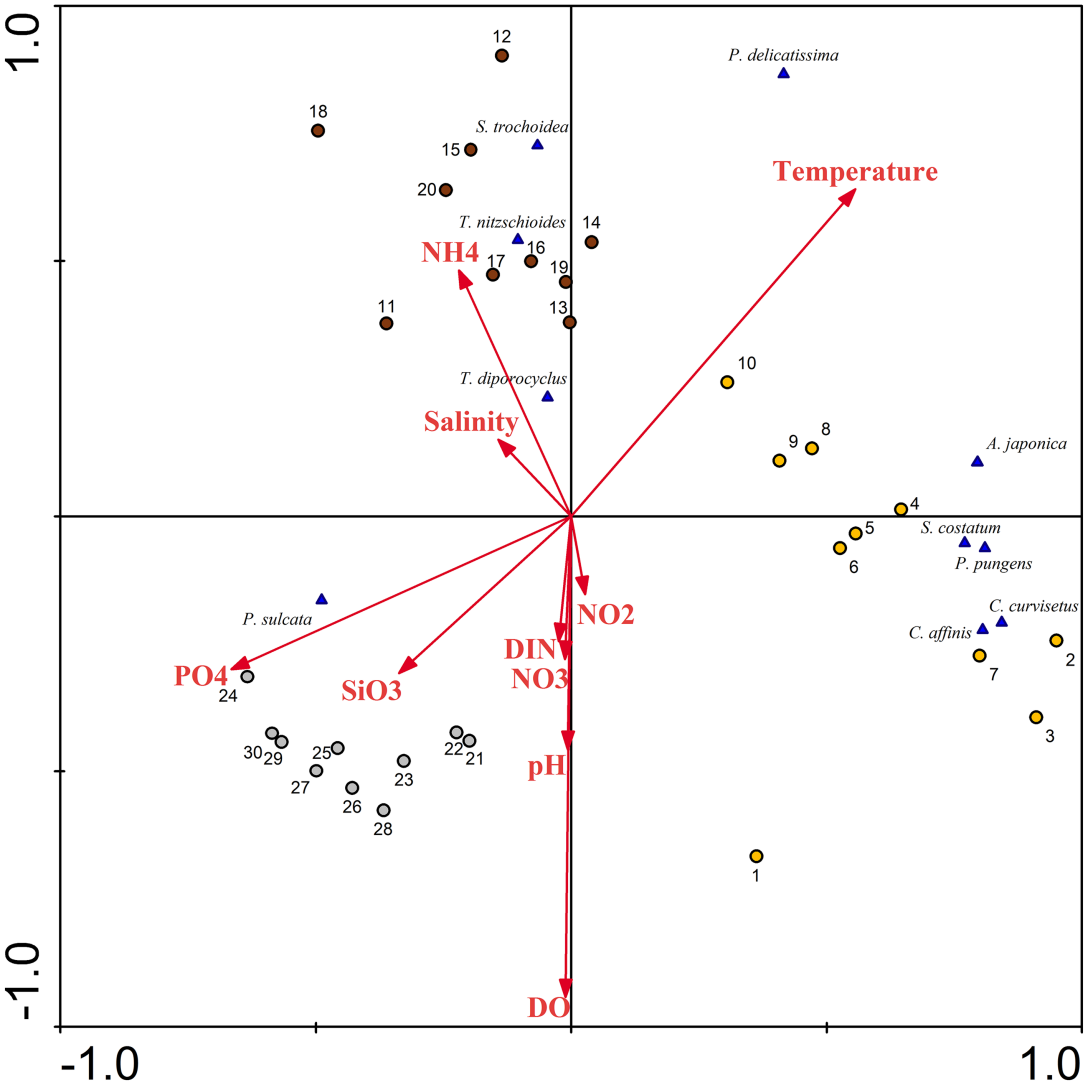
Redundancy analysis (RDA) for phytoplankton abundance with associated chemical parameters in Dongshan Bay (NH_4_: ammonia nitrogen, DIN: dissolved inorganic nitrogen, PO_4_: phosphate SiO_3_: silicate; NO_3_: nitrate; NO_2_: nitrite; DO: dissolved oxygen; the yellow circle represents June; dark red circle represents August; gray circle represents December).

A ranking analysis, based on RDA, was conducted to explore the differences in the spatiotemporal distribution of different phytoplankton species and their corresponding environmental conditions. From June to August (Figure 11), the quadrats of both seasons exhibited a considerably, positive correlation with seawater temperature. This implies that as the seawater temperature in Dongshan Bay continues to rise, the growth rate of phytoplankton accelerates. Consequently, during June and August, there is a higher species diversity and cell abundance of phytoplankton, with various diatoms dominating the phytoplankton community. In essence, the lower water temperature in Dongshan Bay during winter weakens the metabolic activity of phytoplankton cells, leading to lower species diversity and cell abundance. During this season, diatoms such as *M. sulcata* become relatively dominant. Some researchers (Chen et al., 2016; Zhang et al., 2020) indicate that water temperature plays an important role in modulating the diversity of phytoplankton community structure. In our study, the seasonal variations of phytoplankton assemblages were primarily influenced by water temperature. In Dongshan Bay, temperature remains the primary factor influencing the spatiotemporal variability in phytoplankton communities.

The abundance of most diatom species such as *C. curvisetus*, *S. costatum*, and *P. pungens*, was negatively correlated with nutrient concentration. However, the abundance of some diatom species was positively correlated with nutrient concentration. Diatoms can thrive under oligotrophic conditions, potentially due to the presence of heterocystous cyanobacteria living symbiotically with diatoms (Kang and Jia, 2019). This N_2_-fixing cyanobacteria symbionts provide nitrogen for diatoms (Foster et al., 2011). In August, the cell abundance of one dominant dinoflagellates, *S. trochoidea*, showed a considerably negative correlation with phosphate and silicate concentrations. This suggests that this heterotrophic dinoflagellate prefers waters with lower nutrient concentrations. The phytoplankton diversity and distribution in the atoll were primarily associated with nutrients concentration (Jales et al., 2022). Under favorable nutrient conditions, including the presence of dissolved inorganic nitrogen, dissolved inorganic phosphorus and a suitable optimal temperature, phytoplankton exhibited high growth rates throughout the upwelling process from Shanwei to Shantou-Dongshan (Hu et al., 2015). Some chemical factors influenced the distribution of harmful microalgae (Zhang A. et al., 2022). In Dongshan Bay in the present study, nutrients and seawater temperature were the critical factors causing spatiotemporal variability in phytoplankton community structure.

The dominant diatom species, including *T. nitzschioides* and *T. diporocyclus*, demonstrate a noticeable positive correlation with ammonia nitrogen and seawater salinity in the coral habitat area. Thus, phytoplankton could potentially serve as sensitive indicator for discerning habitat differences within coral reef ecosystems.

### 4.3 The response of the phytoplankton community to biotic factors

**Figure 12:**
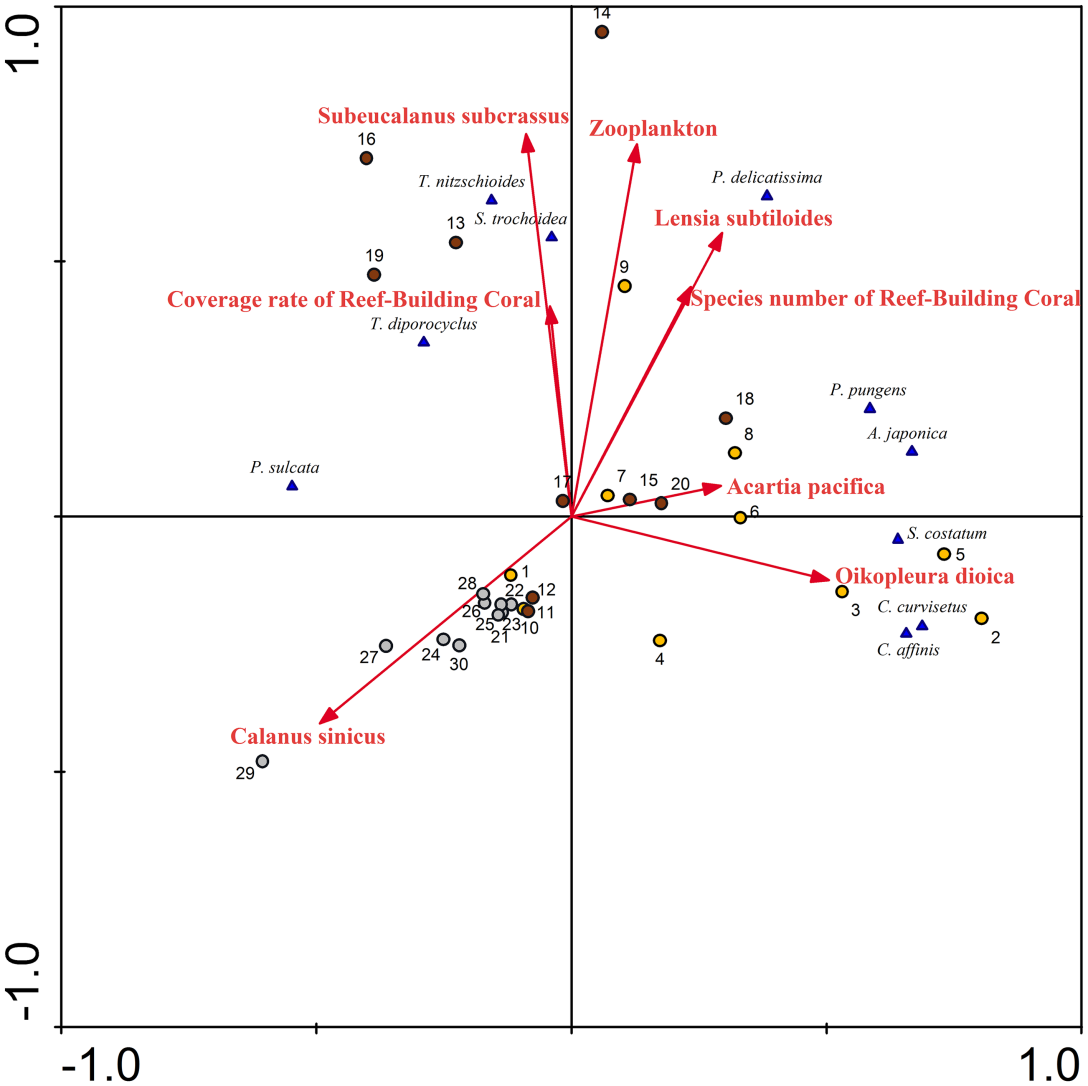
Redundancy analysis (RDA) for phytoplankton abundance with biotic factors, in Dongshan Bay.

In marine ecosystems, zooplankton are major consumers of the primary production of phytoplankton (Ratnarajah et al., 2023) and coral habitats can provide abundant nutrients for phytoplankton (Zheng et al., 2021). The association between predator and prey populations has evolved into a classical and pivotal subject within the natural world (Lampert et al., 1986; Javidi and Ahmad, 2015; Jia et al., 2019; Li et al., 2023). Some research findings indicate that the equilibrium density of phytoplankton populations increases with higher mortality rates, decreased capture rates, and decreased conversion rates, while that of zooplankton increases with an increase in the capture rate (Zhao et al., 2020). The heavily grazing, large zooplankton population probably provides large quantities of phosphate and ammonia that the phytoplankton, suppressed by grazing and now at its annual minimum, do not assimilate. In autumn, the unutilized ammonia might subsequently be oxidized into nitrate (Martin, 1965). Previously, dominant species of phytoplankton have triggered harmful algae blooms along the coast of Fujian, China; these species include *C. curvisetus, S. costatum*, *P. pungens, S. trochoidea*, among others (Zhang, 2022). These species were also the dominant species in Dongshan Bay. Based on the RDA performed on the abundance of phytoplankton species and biotic parameters (Figure 12), it can be concluded that zooplankton density, the coverage rate of reef-building coral, and species number of reef-building coral are positively correlated with the abundance of most dominant species, such as *S. trochoidea, T. nitzschioides*, *T. diporocyclus, S. costatum*, *C. curvisetus*, *C. affinis*, and *P. delicatissima.* The population dynamics of zooplankton is strongly affected by the algal biomass (Lampert et al., 1986). Najmi et al. (2022) have verified that the presence of a substantial amount of phytoplankton is crucial for the survival of zooplankton, and together, they play a vital role in maintaining the sustainability of coral reefs. In coral atolls like the central South China Sea, the south of the Atlantic Ocean, and the Gulf of Aqaba (Red Sea), diatoms and dinoflagellates emerge as the predominant groups (Ke et al., 2018; Jales et al., 2022; Şahin et al., 2022). These findings align with our own research, highlighting the interconnectedness of phytoplankton, zooplankton, and coral reefs. Therefore, we speculate that the abundant distribution of phytoplankton may be one of the factors leading to an increase in the abundance of zooplankton and coral reefs. Due to the thermophilic and photophilic tendencies of predators, as well as their predation, areas with abundant distributions of phytoplankton experience substantial proliferation of zooplankton.

Phytoplankton species such as *S. costatum*, *C. curvisetus*, *C. affinis*, and *P. pungens* negatively correlate with the zooplankton species *C. sinicus*, while they positively correlate with *Acartia pacifica*, and *Oikopleura dioica* (Figure 12). Martin (1965) discovered that plankton tows abundant in *Skeletonema*, *Nitzschia*, *Thalassionema*, and *Chaetoceros* were selectively consumed by copepods (primarily *A. tonsa*), resulting in a swift reduction in *Skeletonema* abundance and the subsequent dominance of *Rhizosolenia* as uneaten cells outgrew other genera. The grazing of zooplankton may be one of the explanations for changes in the phytoplankton community. Some researchers have showed that *Calanus sinicus* predominantly consumes certain harmful algal bloom species, such as *S. costatum*, *C. curvisetus*, *P. pungens*, and *S. trochoidea*. The grazing speed of *C. sinicus* on phytoplankton was food-density dependent, increasing with phytoplankton abundance up to a threshold value and then decreasing regardless of the increase in abundance (Sun et al., 2007). Therefore, in Dongshan Bay in winter, *C. sinicus* exhibits a negative correlation with those harmful algal bloom species.

Phytoplankton grazing is an important component of benthic-pelagic coupling in coral reefs. Smaller copepods, like *Acartia*, show a feeding preference for phytoplankton smaller than 20 μm, exhibiting higher clearance rates (Lee et al., 2012). Since *Skeletonema* and *Chaetoceros* belonging to nano-phytoplankton (2–20 μm) constitute the dominant phytoplankton group in Dongshan Bay, it seems logical to assume that any large *Acartia* population present will utilize these diatoms as its principal food source, leading to heavy grazing of *Skeletonema*, *Chaetoceros*, and *Pseudo-nitzschia*. *Pseudo-nitzschia*, which is often numerically dominant, should also be considered as a phytoplankter that benefits from copepod grazing (Olson et al., 2006). Similarly, during the period of extensive phytoplankton proliferation, a substantial increase in zooplankton such as medusae was observed (Bizani et al., 2023). In our study, *Lensia subtiloides*, which belongs to the medusae, was also positively correlated with some phytoplankton species, such as *T. nitzschioides*, *S. trochoidea*, and *P. delicatissima*. In conclusion, the intricate dynamics of phytoplankton grazing, as evidenced by the feeding preferences of zooplankton and the dominance of specific nano-phytoplankton groups, play a pivotal role in shaping benthic-pelagic coupling in coral habitat.

## 5 Conclusion

This study investigated the dynamics of phytoplankton community responses to environmental factors in Dongshan Bay. The research, conducted in June, August, and December, revealed that the influence of different water currents varies with the seasons. In June, the intrusion of dilute freshwater from the Zhangjiang River estuary resulted in a distinct semi-brackish water phytoplankton community at the bay’s entrance, and the community structure of phytoplankton was notably affected by coastal upwelling. Environmental variables, particularly water temperature and nutrient enrichment, play a considerably role in shaping phytoplankton diversity and community structure. Furthermore, seasonal variations in phytoplankton abundance and their intricate correlations with nutrient concentrations elucidate the interconnected relationship among phytoplankton, zooplankton, and coral community, uncovering how zooplankton density influences specific diatom species. The intricate dynamics of phytoplankton grazing, influenced by zooplankton preferences and the dominance of nano-phytoplankton, emerge as pivotal components in the benthic-pelagic coupling of coral habitat in Dongshan Bay.

## Data availability statement

The raw data supporting the conclusions of this article will be made available by the authors, without undue reservation.

## Author contributions

QZ: Data curation, Formal analysis, Investigation, Software, Writing – original draft. XD: Investigation, Writing – review & editing. JW: Data curation, Project administration, Supervision, Writing – review & editing. YoY: Data curation, Validation, Writing – review & editing. YaY: Data curation, Investigation, Visualization, Writing – review & editing. PX: Writing – review & editing. YC: Funding acquisition, Project administration, Resources, Software, Supervision, Writing – review & editing. XZ: Project administration, Resources, Supervision, Validation, Writing – review & editing.
